# The prevalence and burden of Rome IV bowel disorders of gut brain interaction in patients with non-alcoholic fatty liver disease: a cross-sectional study

**DOI:** 10.1038/s41598-023-35774-5

**Published:** 2023-05-30

**Authors:** Huw Purssell, Lucy Bennett, Oliver Street, Karen Piper Hanley, Neil Hanley, Dipesh H. Vasant, Varinder S. Athwal

**Affiliations:** 1 Department of Gastroenterology and Hepatology, Manchester University NHS Foundation Trust, Manchester, UK; 2 Division of Diabetes, Endocrinology and Gastroenterology, Faculty of Biology, Medicine and Health, University of Manchester, Manchester, UK; 3 NIHR Nottingham Biomedical Research Centre (BRC), Nottingham University Hospitals NHS Trust and the University of Nottingham, Nottingham, UK; 4 Wellcome Trust Centre for Cell-Matrix Research, University of Manchester, Manchester, UK

**Keywords:** Irritable bowel syndrome, Non-alcoholic fatty liver disease

## Abstract

Rome IV bowel disorders of gut brain interaction (DGBI) and non-alcoholic fatty liver disease (NAFLD) are highly prevalent entities with overlapping pathophysiology and risk factors. We aimed to evaluate the prevalence and burden of Rome IV irritable bowel syndrome (IBS) in patients with NAFLD. Patients diagnosed with NAFLD were recruited from a specialist liver clinic. All participants completed assessments to determine liver fibrosis severity, including liver stiffness measurement (LSM), completed the Rome IV diagnostic questionnaire for bowel disorders of gut brain interaction, the IBS symptom severity score (IBS-SSS), and the EQ-5D-5L to measure of quality-of-life (QoL). 142 patients with NAFLD (71 (50%) female, mean age 53.5 (SD ± 14.9), BMI 35.2 (SD ± 8.1) kg/M^2^) were recruited. 79 (55.6%) patients met criteria for a Rome IV bowel DGBI, including 50 patients (35.2%) who met the criteria for IBS (mean IBS-SSS 277.2 (SD ± 131.5)). There was no difference in liver fibrosis scores between those with and without Rome IV IBS (FIB-4 scores *p* = 0.14, LSM *p* = 0.68). Patients with NAFLD and Rome IV IBS had significantly worse QoL scores (EQ-VAS *p* = 0.005 and EQ-5D-5L index *p* = 0.0007), impairment of usual activities of daily living (*p* = 0.012) and were more likely to report anxiety or depression (*p* = 0.038). Rome IV bowel DGBI such as IBS are highly prevalent in patients with NAFLD attending liver clinics and are associated with impaired QoL and psychosocial distress.

## Introduction

Irritable Bowel Syndrome (IBS) and Non-Alcoholic Fatty Liver Disease (NAFLD) are common conditions that present frequently in both primary care and gastroenterology clinics.

NAFLD is characterised by the accumulation of greater than 5% fat in the liver in the absence of an alternative causes (e.g. alcohol, drugs). NAFLD is a spectrum of disease ranging from simple steatosis, through to steatohepatitis, fibrosis and ultimately cirrhosis. The presence of cirrhosis increases the risk of clinically significant events, including hepatocellular carcinoma (HCC), decompensated liver disease and death. It is a major cause of liver disease worldwide with an increasing incidence globally^1^. The population prevalence of NAFLD is estimated to be between 25 and 44%, rising up to a high as 70% in patients with type 2 diabetes^2,3^. In the United Kingdom, liver disease is responsible for the loss of 38,000 and 22,000 working life years, in men and women, respectively^4^. The pathophysiology of NAFLD is associated with the presence of obesity, type 2 diabetes and metabolic syndrome. Genetic risk factors have been shown to increase the likelihood of progressive disease^5,6^.

Irritable Bowel Syndrome (IBS) is a painful, chronic bowel disorder, defined by the Rome IV criteria as a disorder of gut-brain interaction (DGBI) with recurrent abdominal pain and altered bowel habit^7^, with an estimated national prevalence of 4.3% in the United Kingdom^8^. Other Rome IV bowel DGBI including; functional constipation, functional diarrhoea, functional bloating and unspecified Rome IV bowel DGBI, are differentiated from IBS by the presence of the relevant abdominal and bowel symptoms in the absence of pain. Bowel DGBI such as IBS in particular, can be difficult to manage, debilitating and result in significant stigmatisation resulting in increased risk of anxiety and depression and reduced quality of life (QoL)^9–11^.

There is increasing evidence that NAFLD and IBS share overlapping risk factors including obesity, gut microbiome, dietary factors and immune mediated causes^12,13^. Therefore, there has been interest in whether the two conditions co-exist in patient populations. Studies have previously suggested a higher prevalence of IBS in patients with NAFLD compared to global prevalence studies^14,15^. Although the studies have reported the incidence of IBS in NAFLD to be 30%, there have been significant heterogeneity in the diagnostic criteria used for both IBS and NAFLD as well as the populations studied^14,15^. Conversely, studies looking at the prevalence of NAFLD in patients with IBS have also shown a large variability in prevalence, between 3.7% and 74%, with the same disparities in diagnostic criteria usage^16,17^. Moreover, no previous studies have investigated the associations and relationships between the presence of IBS, IBS symptom severity, and liver fibrosis severity in people with NAFLD and co-existing Rome IV IBS.

Our primary aim was to ascertain the prevalence of IBS using Rome IV criteria for bowel DGBI in a population of patients diagnosed with NAFLD at a specialist liver clinic. Our secondary aims were to assess the associations between Rome IV IBS, IBS symptom severity and liver fibrosis using liver stiffness measurements and the association between co-existing IBS and QoL.

## Methods

### Subjects

Patients were recruited as part of the ID LIVER project, a high-throughput assessment pathway for patients with risk factors for liver disease throughout the city of Manchester, United Kingdom (https://sites.manchester.ac.uk/id-liver/). A large proportion of the patients assessed via these specialty clinics are diagnosed with NAFLD, using current best practice criteria^18^.

The study was carried out in accordance with relevant guidelines and regulations. Ethical approval was granted as part of the ID LIVER project by REC North of Scotland (IRAS ID: 273633; REC reference: 20/NS/0055). Patients were recruited to the ID LIVER project prospectively through Liver Assessment Clinics at Manchester University NHS Foundation Trust hospitals between February 2021 and April 2022. Patients were either referred by primary care providers to secondary care specialists for fibrosis assessment following abnormal liver biochemistry/imaging, or identified in the community as having risk factors for liver disease. After informed consent, all patients underwent a thorough clinical history, routine observations including height and weight measurements and a non-invasive aetiological liver screen (alanine aminotransferase, aspartate aminotransferase, alanine amino, full blood count, renal function, thyroid function, fasting lipids, HbA1c, ferritin, transferrin saturations, alpha-1-antitrypsin, autoimmune screen, anti-TTG screen, immunoglobulin profile, viral hepatitis B & C serologies and HIV screen). Non-invasive investigations to determine fibrosis severity were undertaken including liver stiffness measurements using vibration controlled transient elastography (VCTE) and blood-based liver fibrosis scoring systems (FIB-4 score and NAFLD fibrosis score)^19,20^. All patients were asked to complete the EQ-5D-5L questionnaire to assess QoL. An index value was calculated for each patient using the EuroQol EQ-5D-5L index value calculator. Patient responses to the anxiety and depression dimension of the EQ-5D-5L questionnaire were used to assess associations with psychological health.

Exclusion criteria included patients under 18, patients who were pregnant or breast-feeding and patients who had previously known chronic liver disease.

### NAFLD diagnosis and fibrosis assessment

A diagnosis of NAFLD was made by specialist hepatologists in those with imaging suggestive of hepatic steatosis, alcohol consumption below 14 units per week, and a negative non-invasive liver screen excluding alternative aetiologies, in line with national guideline^18^. FIB-4 and NAFLD fibrosis score were calculated using published formulas^19,20^. Nationally recognised cut offs were used to stage fibrosis^18^. Liver Stiffness measurements (LSM) were recorded using Fibroscan 530 (Echosens, Paris, France) with either M or XL probes on all patients seen in the Liver Assessment Clinics. Fibroscan was performed by doctors and specialist nurses with appropriate training. LSM were reported in median kPa over 10 readings with an interquartile range less than 30%. Controlled Attenuation Pressure (CAP) results were collected to non-invasively assess hepatic steatosis.

### Rome IV bowel disorders of gut brain interaction and symptom severity

Patients diagnosed with NAFLD were asked to complete the Rome IV diagnostic questionnaire for Rome IV bowel DGBI (IBS, Functional Constipation, Functional Diarrhoea, Functional Bloating, or Unspecified Rome IV bowel DGBI)^21^. All participants also completed the IBS Symptom Severity Score (IBS-SSS) as a measure of bowel symptom severity^22^. The IBS-SSS score records frequency and severity of abdominal pain and distention as well as an individual’s satisfaction with their bowels and its impact on daily life. Cut offs of < 150, 150–300 and > 300 (maximum score is 500) are widely used to classify IBS symptoms into mild, moderate and severe categories.

### Statistical analysis

Data on the co-existing prevalence and burden of having a Rome IV bowel DGBI including Rome IV IBS, liver fibrosis and IBS severity, were compared using Student t-tests, Mann Whitney U-test and Chi-squared tests. The EQ-VAS score was used to measure QoL and was analysed using Student t-tests. Patient responses to the anxiety and depression sub-scores of the EQ-VAS were analysed using a Chi-squared test. All data was analysed using Prism version 9.0 (GraphPad, USA) and Microsoft Excel (Microsoft, USA).

## Results

### Patient demographics

**Figure 1:**
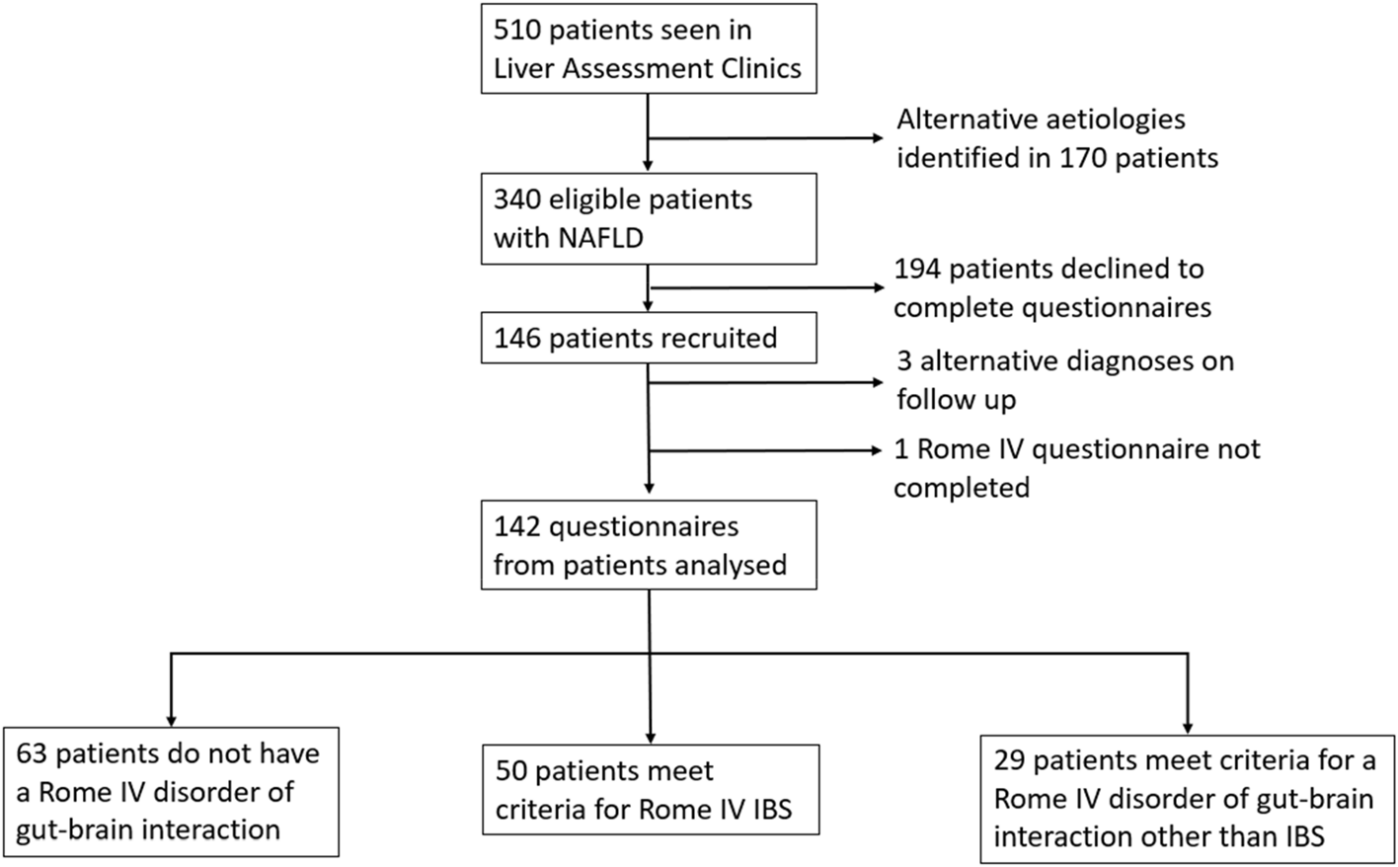
Flowchart of patient identification and recruitment.

From 340 potentially eligible patients diagnosed with NAFLD, 146 (43%) patients were recruited and completed the Rome IV diagnostic questionnaire and IBS-SSS questionnaire. 3 patients were subsequently found to have an alternative liver diagnosis on follow-up consultation (1 diagnosis of hereditary haemochromatosis, 1 diagnosis of Primary Biliary Cholangitis and 1 diagnosis of seronegative autoimmune hepatitis) and were therefore excluded. One questionnaire was uninterpretable and excluded from analysis (Fig. 1). 142 patients were therefore included in the study. Table 1 shows the characteristics of recruited patients, those with Rome IV IBS and those without. There were equal numbers of male and female participants with a mean age of 53.5 (standard deviation (SD) ± 14.9) years. Those with Rome IV IBS tended to be younger in age (*P* = 0.05, Table 1). The mean BMI was 35.2 (SD ± 8.1) kg/m^2^ and mean alcohol consumption was 4.6 (SD ± 10.5) units/week. 58 (40.8%) patients had type 2 diabetes. 48 (33.6%) patients were prescribed metformin. All patients had a negative coeliac serology.

**Table 1:** Patient characteristics in the whole cohort, patients meeting meeting Rome IV criteria for IBS and those who do not.

	Total cohort	Rome IV IBS	No IBS	*P* value (95% CI)
Number of patients	142	50 (35.2%)	92 (64.8%)	
Age (years)	53.5 ± 14.9	50.2 ± 15.3	55.3 ± 14.5	0.053 (− 0.08 to 10.20)
Male	71 (50%)	22 (31.0%)	49 (69.0%)	0.380
Female	71 (50%)	28 (39.4%)	43 (60.6%)	
Waist circumference (cm)	116.3 ± 16.9	118.0 ± 14.8	115.4 ± 17.97	0.378 (− 8.53 to 3.26)
BMI	35.2 ± 8.1	35.7 ± 6.7	35.0 ± 8.8	0.639 (− 3.49 to 2.15)
Type 2 diabetes	58 (40.8%)	17 (34%)	41 (44.6%)	0.284
Alcohol units/week	4.6 ± 10.5	3.5	5.3	0.340 (− 1.87 to 5.39)
Prior diagnosis of IBS	11 (7.7%)	8 (16%)	3 (3.3%)	
CAP steatosis score	302.5 ± 71.2	311.3 ± 64.8	297.8 ± 74.3	0.387 (− 38.67 to 11.55)
IQR/Median	12.1 ± 4.9	12.5 ± 4.5	11.9 ± 5.1	0.50 (− 2.31 to 1.13)
ALT (IU)	55.7 ± 44.1	49.4 ± 29.1	59.1 ± 50.2	0.212 (− 5.65 to 25.20)
AST (IU)	40.7 ± 24.7	36.2 ± 16.8	43.2 ± 27.9	0.118 (− 1.82 to 15.80)
ALP (IU)	97.5 ± 45.7	95.6 ± 43.7	98.5 ± 46.9	0.717 (− 13.10 to 18.99)
Gamma GT (IU)	76.5 ± 110.1	78.8 ± 80.7	75.3 ± 123.4	0.859 (− 42.63 to 35.61)
HbA1c (mmol/mol)	46.6 ± 14.7	46.3 ± 14.7	46.7 ± 14.8	0.858 (− 4.78 to 5.73)
EQ-5D-5L index	0.75 ± 0.30	0.64 ± 0.32	0.81 ± 0.25	***0.0007 (0.07 to 0.27)
EQ VAS	68.6 ± 21.6	60.9 ± 20.8	71.8 ± 22.2	**0.005 (3.28 to 18.62)
IBS-SSS	148.7 ± 131.5	277.2 ± 103.1	78.2 ± 83.1	*** < 0.001 (− 230.6 to − 160.5)

Parametric data is presented as mean ± standard deviation (SD)). Non-parametric data is presented as mean (% of cohort). **p < 0.01, ***p < 0.001.

### Prevalence of Rome IV bowel disorders of gut brain interaction in NAFLD

**Figure 2:**
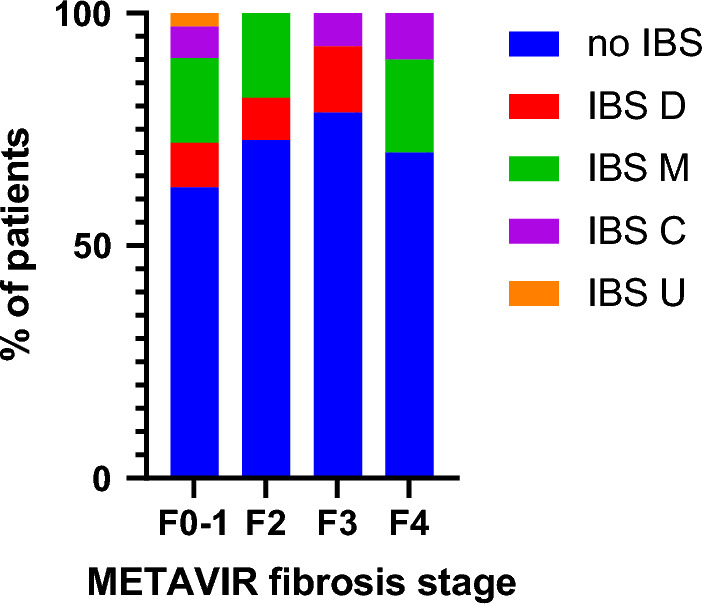
Bar graph showing the percentage of patients with each subtype of IBS for each METAVIR fibrosis stage determined by liver stiffness measurements.

79 (55.6%) patients met criteria for a Rome IV DGBI and 50 (35.2%) patients in our cohort had Rome IV IBS. However, only 11/142 (7.7%) patients already had a prior clinical diagnosis of IBS. In those with Rome IV IBS, the most common subtypes were mixed-IBS (24/50 patients (48%)) and diarrhoea-predominant IBS (14/50 patients (28%)). Whereas, 9 (18%) patients had constipation-predominant IBS, and 3 (6%) had unclassified IBS. Figure 2 shows the percentage of patients with each IBS subtype split by METAVIR fibrosis stage determined by LSM. The prevalence of Rome IV IBS in this cohort of patients with NAFLD was similar in males and females (31.0% vs. 39.4%, Table 1). There were also no differences in mean age, BMI, waist circumference, or the presence of type 2 diabetes between patients with and without Rome IV IBS, and similar mean ALT, AST, ALP, Gamma GT, HbA1c and Ferritin levels (Table 1).

In terms of IBS severity, 24/50 (48%) patients meeting criteria for Rome IV IBS experienced moderate symptoms, and 19 (38%) had severe symptoms based on the IBS-SSS questionnaire^22^. The mean symptom severity in those with IBS was 277.2 (SD ± 103.1).

29 (20.4%) patients did not meet criteria for IBS, however, did meet criteria for another Rome IV bowel DGBI. 10/29 (34.4%) of these had functional constipation and 8/29 (27.6%) patients met criteria for functional diarrhoea. 8/29 (27.6%) patients had an unspecified Rome IV bowel DGBI, and 3/29 (10.3%) met criteria for functional bloating. Sub-analysis of patients meeting criteria for any Rome IV bowel DGBI found that there was no differences in LSM (8.1 kPa vs. 7.5 kPa (*p* = 0.51; 95% CI − 2.51–1.25)), FIB-4 score (1.36 vs. 1.18 (p = 0.19; 95% CI − 0.09–0.44)) or NAFLD fibrosis score (− 0.82 vs. − 1.33; 95% CI − 0.08–1.09)). There were no significant differences noted in hepatic steatosis measured by controlled attenuation pressure (303.7 dB/m vs. 300.9 dB/m (*p* = 0.82; 95% CI − 26.97–21.26)).

Presence of any Rome IV bowel DGBI was associated with a significantly worse quality of life measured by EQ-VAS score than those without (76.1 vs. 62.2 (*p* = 0.0001; 95% CI 6.9–20.78). However, anxiety and depression levels did not significantly differ (*p* = 0.11).

### Rome IV IBS and NAFLD liver fibrosis severity

The median LSM across the whole cohort was 6.3 (SD ± 5.7) kPa. There were no significant differences in liver stiffness measurements between patients with IBS and those without (8.1 kPa vs. 7.7 kPa (*p* = 0.68; 95% CI − 2.38–1.56)). There were no significant differences in steatosis assessment by controlled attenuation pressure (311.3 dB/m vs. 297.8 dB/m (*p* = 0.29; 95% CI − 38.67–11.55)), FIB-4 score (1.13 vs. 1.34 (*p* = 0.15; 95% CI − 0.07–0.48) or NAFLD Fibrosis score (− 0.89 to − 1.46 (*p* = 0.064; 95% CI − 0.034–1.179) between patients with IBS and those without. Within the 50 patients identified with Rome IV IBS, there was no correlation between the IBS-SSS scores and LSM (r^2^ = 0.03), CAP (r^2^ = 0.0002), FIB-4 score (r^2^ = 0.02) or NAFLD fibrosis score (r^2^ = 0.007).

### The burden of co-existing Rome IV IBS and NAFLD

**Figure 3:**
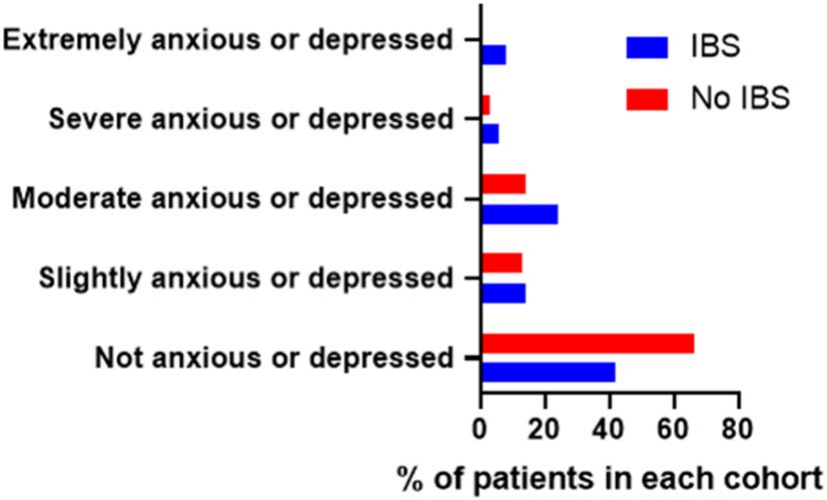
Bar graph plots showing self-reported patient responses regarding anxiety or depression on EQ-5D-5L questionnaire.

**Figure 4:**
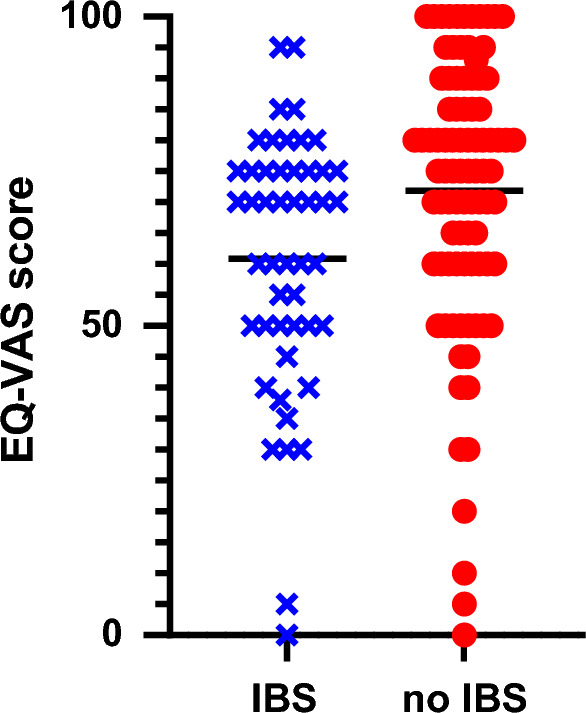
Scatter plot showing the differences in EQ-VAS responses for quality of life in patients meeting Rome IV criteria for IBS and those not. The mean EQ VAS score for patients with IBS was 62.2 whilst patients without IBS had a mean EQ VAS of 71.8 (*p* = 0.006; 95% CI 3.27–18.62).

Overall, 5/142 (3.5%) patients did not complete the EQ-5D-5L questionnaire. Amongst the 137 patients that completed the EQ-5D-5L questionnaire, those with Rome IV IBS reported significantly worse global assessment of health using EQ-VAS score and EQ-5D-5L questionnaires, compared to patients without Rome IV IBS (Table 1). Patients with Rome IV IBS were also more likely to experience anxiety and depression symptoms and poorer QoL (Table 1, Figs. 3 and 4). In addition, patients with Rome IV IBS were more likely to have difficulties with their usual activities (*p* = 0.012), and impairment due to pain and discomfort (*p* = 0.013), compared to those without IBS (supplementary Table 1).

## Discussion

This study demonstrates that symptoms compatible with a Rome IV bowel DGBI are highly prevalent, affecting over 50% of people diagnosed with NAFLD. Most of these patients fulfilled Rome IV IBS criteria, the most severe bowel DGBI. The prevalence of Rome IV IBS in our cohort of patients with NAFLD in the liver assessment clinic was 35.2%; nearly ten times the published prevalence of IBS in global and UK populations, based on an estimated Rome IV IBS prevalence of 4.3% in the UK ^8^.

Importantly, our findings corroborate previous studies reporting a prevalence of IBS in patients with NAFLD of 29.2% and 29.4% respectively^14,15^. Our findings are also consistent with recent data suggesting that patients with NAFLD are significantly more likely to develop IBS than those without NAFLD^23^. In contrast to epidemiological data in general populations where IBS is estimated to affect approximately 1.5 times more females than males^24^, our data in a NAFLD population did not show a significant difference in the prevalence of Rome IV IBS between males and females with NAFLD. This disparity most likely reflects the demographics of the NAFLD population studied, rather than sex-specific differences in the pathophysiology of IBS in patients with NAFLD.

Interestingly, a further twenty percent of patients in our study who did not meet the Rome IV criteria for IBS, met the criteria for other, less severe, Rome IV bowel DGBI which were also associated with significant impairment of QoL.

Similar to previous studies of patients with NAFLD and chronic diarrhoea^25^, there was no association between the presence of Rome IV IBS and liver stiffness measurements in our study. Moreover, there were no associations between IBS symptom severity, liver stiffness measurements, blood-based fibrosis assessments or steatosis measurements.

Despite the high prevalence of IBS in our study, only 16% of patients with Rome IV IBS had a prior diagnosis of IBS. This suggests that there is a large proportion of patients with undiagnosed IBS attending liver clinics with NAFLD, often with severe symptoms. The importance of this is that 86% of those with NAFLD who met the Rome IV criteria for IBS had moderate-to-severe symptoms. This degree of IBS symptom severity seen in the liver clinic is above the UK national average^8^ and is similar to that seen in tertiary IBS clinics^26^. As far as the authors are aware, none of the previous literature describing the incidence of IBS in NAFLD used any validated symptom severity scores for IBS. Indeed, our data do suggest that Rome IV IBS is associated with considerable morbidity with a significantly worse QoL, impaired ability to perform activities of daily living, and an increased incidence of severe anxiety and depression, compared to patients with NAFLD without IBS. This replicates findings from Jones-Pauley et al.^14^ and it is well documented that IBS is associated with increased levels of anxiety and depression^11^. Troublesome symptoms such as diarrhoea and incontinence can be distressing for patients and difficult to manage medically leading to frustration and a reduced QoL^9,27^. This study will therefore be important in raising awareness of the burden of overlapping Rome IV bowel DGBI and NAFLD in patients presenting to liver clinics.

Recent evidence-based clinical guidelines on bowel DGBI place an emphasis on making a positive diagnosis of IBS in the absence of alarm features, and also provide guidance on which patients would benefit from targeted investigations to exclude IBS mimics including coeliac disease, microscopic colitis, and bile salt malabsorption^28^. The findings of our study suggest that patients with NAFLD may not always be forthcoming about their bowel symptoms. The findings are therefore likely to be of importance to both luminal gastroenterologists and hepatologists looking after people with these conditions. This study suggests that screening for bowel DGBI in patients with NAFLD attending liver clinics has the potential to allow a positive diagnosis, adjust any medications (e.g. metformin) which may contribute to symptoms, order relevant tests to exclude mimics, and then proceed with holistic symptomatic treatments including dietary, medical and behavioural approaches to improve bowel symptoms.

As far as the authors are aware, this is the first study investigating the overlap of the entire spectrum of Rome IV bowel DGBI to include liver fibrosis assessment using liver stiffness measurements to determine liver disease severity, as well as a validated instrument to assess IBS symptom severity. However, there are several limitations to our study. Firstly, patients in ID LIVER hepatology assessment clinics did not undergo luminal gastroenterology evaluations to exclude IBS mimics other than coeliac disease. Reassuringly, however, all patients included had a negative coeliac serology as part of their autoimmune screen, and a structured past medical and surgical history was taken to exclude red flags and none of those included had any known organic gastrointestinal diagnoses. However, in the sub-set of patients with diarrhoea predominant symptoms (22/79 (27.8%) patients) with a Rome IV bowel disorder of gut brain interaction (functional diarrhoea or diarrhoea predominant IBS), it is recognised that around a third of patients would benefit from investigations to exclude bile salt malabsorption^28^, and up to 8% may have microscopic colitis^29^. Secondly, we cannot exclude responder bias. Only 146/340 (43%) of patients screened with NAFLD agreed to complete the bowel questionnaires. Those with significant bowel symptoms may have been more motivated to complete the bowel questionnaires than those without symptoms. One reason for the lower recruitment rate, and a potential source of accrual bias, is that some patients were unable to complete questionnaires due to language barriers (the questionnaires were only available in English). Thirdly, NAFLD was frequently found incidentally as part of investigations for other abdominal symptoms and hence referred into clinic. It is therefore possible that more symptomatic patients fulfilling Rome IV IBS were included in our study compared to the situation if patients were identified through population screening. However, the lack of pre-existing organic gastrointestinal diagnoses in our population, and the fact the only a minority of those with Rome IV IBS had a prior IBS diagnosis, suggests that most patients had not had their significant bowel symptoms recognised by a healthcare provider which is an important finding given the association with a worse QoL and higher reported psychological distress. Finally, to minimise questionnaire fatigue and improve study completion amongst participants of the ID LIVER project, a dedicated validated instrument for anxiety and depression such as the hospital anxiety and depression scale was not included. Whilst it was notable that anxiety or depression was reported more frequently in those with Rome IV IBS on the EQ-5D-5L questionnaire, future studies should investigate this further using specific anxiety and depression questionnaires.

To further investigate the co-existence of IBS in NAFLD, further large-scale cohort studies would be needed with targeted investigations, where appropriate, to exclude IBS mimics.

## Conclusion

Debilitating symptoms from Rome IV bowel DGBI are extremely common, affecting over half of those attending liver clinics with NAFLD. These are a significant contribution to the morbidity in people with NAFLD. Whilst bowel symptom severity does not appear to be associated with the degree of liver fibrosis, the overlapping bowel symptoms are often severe enough to be associated with a worse QoL, impairments in activities of daily living, and psychological distress. Physicians should therefore be aware of the relationship, as early intervention with targeted investigations to exclude IBS mimics and symptomatic treatments may reduce morbidity and improve QoL.

## Supplementary Information

Supplementary Information.

## Data Availability

The anonymised datasets generated during the study are available from the corresponding author on reasonable request.
