# Supplementary material for: The prevalence and burden of Rome IV bowel disorders of gut brain interaction in patients with non-alcoholic fatty liver disease: a cross-sectional study
Source: Sci Rep. 2023 May 30;13:8769. doi: 10.1038/s41598-023-35774-5 (PMC10229668; doi:10.1038/s41598-023-35774-5)
Supplement: Supplementary file 1 — Supplementary Information. [file 41598_2023_35774_MOESM1_ESM.docx]

| **Dimension** | **Patients with Rome IV IBS (n=47)** | **Patients without Rome IV IBS (n=90)** | **P value** |
| --- | --- | --- | --- |
| **Mobility** | | | |
| No problems | 25 (53.2%) | 66 (73.3%) | 0.090 |
| Slight problems | 10 (21.3%) | 12 (13.3%) |  |
| Moderate problems | 9 (19.1%) | 7 (7.8%) |  |
| Severe problems | 3 (6.4%) | 5 (5.6%) |  |
| Unable to walk about | 0 | 0 |  |
| **Self Care** | | | |
| No problems | 37 (78.7%) | 80 (88.9%) | 0.110 |
| Slight problems | 3 {6.4%) | 5 (5.6%) |  |
| Moderate problems | 6 (12.8%) | 3 (3.3%) |  |
| Severe problems | 0 (0%) | 2 (2.2%) |  |
| Unable to wash or dress | 1 (2.1%) | 0 |  |
| **Usual activities** | | | |
| No problems | 25 (53.2%) | 73 (81.1%) | **0.012** |
| Slight problems | 11 (23.4%) | 7 (7.8%) |  |
| Moderate problems | 7 (14.9%) | 5 (5.6%) |  |
| Severe problems | 2 (4.3%) | 2 (2.2%) |  |
| Unable to do usual activities | 2 (4.3%) | 3 (3.3%) |  |
| **Pain/Discomfort** | | | |
| No pain/discomfort | 15 (31.9%) | 52 (57.8%) | **0.013** |
| Slight pain/discomfort | 10 (21.3%) | 19 (21.1%) |  |
| Moderate pain/discomfort | 13 (27.7%) | 10 (11.1%) |  |
| Severe pain/discomfort | 6 (12.8%) | 8 (8.9%) |  |
| Extreme pain/discomfort | 3 (6.4%) | 1 (1.1%) |  |
| **Anxiety & Depression** | | | |
| Not anxious or depressed | 21 (44.7%) | 61 (67.8%) | **0.038** |
| Slightly anxious or depressed | 7 (14.9%) | 12 (13.3%) |  |
| Moderately anxious or depressed | 12 (25.5%) | 13 (14.4%) |  |
| Severely anxious or depressed | 3 (6.4%) | 3 (3.3%) |  |
| Extremely anxious or depressed | 4 (8.5%) | 1 (1.1%) |  |

Supplementary Table 1: Completed EQ-5D-5L results for patients with Rome IV IBS and those without. Statistical significance across all proportions performed using Chi Squared test. 5 patients (3 with Rome IV IBS, 2 without IBS) did not complete questionnaire.
